# Mindfulness as a mediator between intolerance of uncertainty and test anxiety among adolescents: a structural equation model

**DOI:** 10.3389/fpsyg.2026.1793275

**Published:** 2026-04-08

**Authors:** Ahmet Sapanci, Hamdi Korkman, Mark D. Griffiths

**Affiliations:** 1 Department of Psychological Counseling and Guidance, Düzce University, Düzce, Türkiye; 2 Department of Social Work, Sandıklı School of Applied Sciences, Afyon Kocatepe University, Afyonkarahisar, Türkiye; 3 Afyon Kocatepe Universitesi, Afyonkarahisar, Türkiye; 4 International Gaming Research Unit, Department of Psychology, Nottingham Trent University, Nottingham, United Kingdom

**Keywords:** Acceptance and Commitment Therapy, adolescents, Attentional Control Theory, intolerance of uncertainty, mindfulness, test anxiety

## Abstract

Test anxiety is a prevalent problem among adolescents and is closely associated with cognitive and emotional responses to uncertainty. Although intolerance of uncertainty and mindfulness have each been linked to anxiety, their joint role within a unified explanatory model remains insufficiently explored. The present study examined whether mindfulness mediates the relationship between intolerance of uncertainty and test anxiety among adolescents in Türkiye. Using a cross-sectional correlational design, data were collected between March and April 2025 from 834 high school students (aged 14–18 years) recruited through convenience sampling from multiple public secondary schools located in the city center of Afyon, Türkiye. Participants completed standardized self-report measures assessing intolerance of uncertainty, mindfulness, and test anxiety. Structural equation modeling was conducted using LISREL 8.8, following preliminary analyses in SPSS 27. Structural equation modeling showed that higher levels of intolerance of uncertainty were associated with lower mindfulness, which in turn predicted higher levels of test anxiety, supporting a partial mediation model. These findings identify mindfulness as a significant mediating variable in the association between intolerance of uncertainty and test anxiety and contribute to the understanding of their interrelations within evaluative academic contexts.

## 1 Introduction

Uncertainty is an inherent feature of academic life. However, when perceived as uncontrollable and threatening, it becomes a significant psychological stressor. From an evolutionary perspective, unpredictability has historically signaled potential danger, activating expectancy-based vigilance and defensive responses (Carleton, 2016). Although contemporary academic environments no longer involve physical threats, evaluative uncertainty continues to elicit threat-related cognitive and emotional reactions. In centralized and performance-based educational systems where examination outcomes determine academic tracking and future opportunities, uncertainty becomes not merely situational but structurally consequential (Organization for Economic Co-operation Development [OECD], 2021).

For adolescents, this institutionalized uncertainty carries particular psychological urgency. Developmentally, adolescence is characterized by heightened sensitivity to social evaluation, identity formation, and future-oriented concerns. Within high-stakes examination contexts, ambiguity surrounding performance outcomes may generate persistent anticipatory worry, intrusive rumination, sleep disturbances, and heightened physiological arousal. Such processes render test anxiety a developmentally salient and psychologically consequential phenomenon.

At the dispositional level, intolerance of uncertainty (IU) refers to a generalized cognitive-affective tendency to perceive ambiguous situations as aversive and threatening (Carleton, 2016). Transdiagnostic frameworks position IU as a central vulnerability factor implicated in anxiety- and stress-related psychopathology (McEvoy and Erceg-Hurn, 2016; Shihata et al., 2016; Tanovic et al., 2018). Experimental and psychophysiological evidence indicates that individuals high in IU exhibit heightened threat expectancy, difficulty disengaging from ambiguity-related cues, and reduced attentional inhibition under uncertain conditions (Morriss and McSorley, 2019; Tanovic et al., 2018). Within evaluative academic contexts, such cognitive tendencies may intensify catastrophic interpretations of exam-related ambiguity and disrupt performance through attentional interference.

Conceptually, intolerance of uncertainty reflects a broad dispositional sensitivity to ambiguity across life domains, whereas test anxiety represents domain-specific evaluative distress emerging in performance-based academic settings. Although traditional models of test anxiety emphasize situational stressors and emotional arousal (Liebert and Morris, 1967; Wine, 1971), they offer comparatively limited specification regarding how enduring dispositional vulnerabilities become translated into evaluative distress. Contemporary cognitive-affective perspectives suggest that anxiety in evaluative contexts is shaped not only by external demands but also by individuals’ regulatory relationship with their internal experiences.

Within this theoretical shift, Acceptance and Commitment Therapy (ACT) posits that psychological distress is maintained by rigid and avoidant responses to internal states (Hayes et al., 1999), whereas Attention Control Theory (AtCT) suggests that anxiety reallocates cognitive resources toward threat-related processing at the expense of goal-directed performance (Eysenck et al., 2007). IU-related threat sensitivity may increase cognitive fusion and threat-focused attentional allocation—processes that mindfulness directly targets by fostering decentering and attentional flexibility. Integrating these perspectives highlights mindfulness as a regulatory process potentially linking dispositional uncertainty sensitivity to evaluative anxiety.

Mindfulness is defined as present-focused, nonjudgmental awareness of internal experiences (Bishop et al., 2004). By enhancing attentional stability and reducing experiential avoidance, mindfulness supports cognitive flexibility and attenuates threat-driven attentional bias (Gu et al., 2015; Lindsay and Creswell, 2017; Tang et al., 2015). Empirical findings indicate that mindfulness is negatively associated with academic worry and stress reactivity among adolescents (Quach et al., 2016), and meta-analytic evidence demonstrates that mindfulness-based interventions significantly reduce test anxiety among student populations (Dvořáková et al., 2017; Yılmazer et al., 2024). These findings suggest that mindfulness may function not merely as a protective correlate, but as a theoretically coherent process-level pathway through which IU-related vulnerability becomes associated with evaluative distress.

Importantly, few studies have simultaneously examined intolerance of uncertainty, mindfulness, and test anxiety within a theoretically articulated structural mediation framework among adolescents. Existing research has largely relied on correlational approaches or partial models, leaving the indirect pathway linking dispositional uncertainty sensitivity to evaluative anxiety insufficiently specified. Clarifying this process is particularly relevant in high-stakes academic contexts where uncertainty-related distress may undermine both academic functioning and psychological wellbeing.

The present study addresses this empirical gap by proposing and testing a process-based structural equation model in which mindfulness mediates the relationship between intolerance of uncertainty and test anxiety. Mindfulness is conceptualized as a regulatory capacity that may be shaped by dispositional tendencies such as IU, rather than as a situational buffer that merely moderates stress responses. This conceptualization provides theoretical justification for evaluating a mediation model. Although the proposed framework specifies directional paths consistent with contemporary cognitive-affective theory, the study does not assume causal inference; rather, it examines whether the observed data are consistent with a theoretically grounded process pathway. Drawing on the theoretical and empirical literature reviewed above, it was hypothesized that:

- Intolerance of uncertainty (IU) would be positively associated with test anxiety (H_1_).
- Intolerance of uncertainty (IU) would be negatively associated with mindfulness (H_2_).
- Mindfulness would be negatively associated with test anxiety (H_3_).
- Mindfulness would mediate the relationship between intolerance of uncertainty (IU) and test anxiety, such that IU would exert a significant indirect effect on test anxiety through mindfulness (H_4_).

## 2 Materials and methods

### 2.1 Study design

The present study utilized a correlational research design, which systematically examined the statistical relationships between two or more variables without implying causality (Creswell and Creswell, 2018). Correlational designs are particularly suitable for exploring relational patterns among psychological constructs in naturalistic settings. The present study investigated whether mindfulness functioned as a mediating variable in the relationship between individuals’ levels of IU and their levels of test anxiety. To examine both direct and indirect effects simultaneously, structural equation modeling (SEM) was employed. SEM is a robust statistical technique that enables researchers to test complex theoretical assumptions by analyzing relationships among both observed and latent variables (Schumacker and Lomax, 2016). In the proposed model, IU was specified as the independent variable, test anxiety was the dependent variable, and mindfulness was the mediating variable.

### 2.2 Participants

The sample comprised 834 high school students enrolled in various types of public secondary schools located in the city center of Afyon, Türkiye [71.8% female (*n* = 599) and 28.2% male (*n* = 235)]. With respect to school type, the majority of the students were attending Anatolian high schools (54.0%), followed by science high schools (17.7%), religious vocational high schools (15.8%), vocational and technical high schools (11.5%), and social sciences high schools (1.0%). This distribution indicates a heterogeneous sample comprising students from diverse educational backgrounds, thereby enhancing the generalizability of the findings across different high school settings.

High school students were selected as the target population because adolescence represents a developmental period characterized by heightened sensitivity to uncertainty and evaluative stress. In the Turkish educational context, high-stakes national examinations substantially influence academic and professional trajectories, rendering exam-related anxiety a salient and ecologically valid construct within this age group. Moreover, intolerance of uncertainty and attentional regulation processes undergo significant developmental refinement during adolescence, making this population theoretically appropriate for testing the proposed mediation model.

Eligibility criteria required participants to (i) be currently enrolled in high school, (ii) fall within the typical adolescent age range (14–18 years), and (iii) provide informed consent. Cases were excluded if the surveys contained substantial missing data (greater than 10%), demonstrated patterned or careless responding, or fell outside the specified age range. After data screening procedures, the final analytic sample comprised 834 participants.

### 2.3 Measures

#### 2.3.1 Intolerance of Uncertainty Scale

The 12-item IUS-12 (Carleton et al., 2007; Turkish version: Sarıçam et al., 2014) was used to assess individuals’ cognitive, emotional, and behavioral responses to uncertain situations and is the brief version of the original 27-item scale (Freeston et al., 1994). The scale assesses two factors: prospective anxiety (e.g., anxiety about future uncertainty) and inhibitory anxiety (e.g., behavioral inhibition in the face of uncertainty). Items (e.g., *“Unforeseen events upset me greatly”* and *“I feel uncomfortable in unplanned situations”*) are rated on a five-point scale ranging from 1 (*not at all characteristic of me*) to 5 (*entirely characteristic of me*). The total scores range from 12 to 60, with higher scores indicating greater intolerance of uncertainty. In the present study, the internal consistency was very good (Cronbach’s alpha = 0.87).

#### 2.3.2 Mindful Attention Awareness Scale

The 15-item MAAS (Brown and Ryan, 2003; Turkish version: Özyeşil et al., 2011) was used to assess the extent to which individuals attend to and are aware of present-moment experiences in their daily lives. Items (e.g., *“I find it difficult to stay focused on what’s happening in the present”* and *“I do things automatically without being aware of what I’m doing”*) are rated on a 6-point Likert scale ranging from 1 (*almost always*) to 6 (*almost never*). The total scores range from 15 to 90, with higher scores indicating greater levels of mindfulness. In the present study, the internal consistency was very good (Cronbach’s alpha = 0.87).

#### 2.3.3 Westside Test Anxiety Scale

The 11-item WTAS (Driscoll, 2007; Turkish version: Totan and Yavuz, 2009) was used to assess students’ levels of anxiety specifically related to exam settings. Items (e.g., *“As an important exam approach, I find it harder to concentrate on studying”* and *“During important tests, I may forget what I know owing to lack of focus”*) are rated on a five-point scale ranging from 1 (*not at all true*) to 5 (*always true*). Total scores range from 11 to 55, with higher scores indicating greater levels of test anxiety. In the present study, the internal consistency was excellent (Cronbach’s alpha = 0.90).

To enhance methodological transparency and address concerns regarding indicator specification, Table 1 presents a detailed summary of the latent constructs, their observed indicators (named parcels), psychological representations, instruments, item counts, and validation references. In line with recommendations for structural equation modeling, parceling was employed for three primary reasons: (i) to reduce item-level random measurement error, (ii) to improve indicator reliability and model parsimony, and (iii) to provide a more stable representation of complex cognitive-affective constructs in adolescents.

**TABLE 1:** Summary of study variables, named parcels, psychological representations, and instruments.

Latent variable	Observed indicators (named parcels)	Psychological representation	Instrument	Number of items	References
Intolerance of uncertainty	Prospective anxiety; inhibitory anxiety; uncertainty reactivity	Threat anticipation, behavioral inhibition, and heightened reactivity under ambiguity	IUS-12	12	(Carleton et al., 2007; Sarıçam et al., 2014)
Mindfulness	Attentional awareness; present-moment focus; non-automatic responding	Present-moment attentional regulation and cognitive flexibility capacity	MAAS	15	(Brown and Ryan, 2003; Özyeşil et al., 2011)
Test anxiety	Cognitive worry; physiological arousal; impaired concentration	Exam-related worry, autonomic arousal, and disruption of attentional control	WTAS	11	(Driscoll, 2007; Totan and Yavuz, 2009)

Parcels were used as observed indicators in the measurement model. Each parcel reflects theoretically coherent subcomponents consistent with the original factor structures of the instruments.

Because each latent construct was theoretically coherent and demonstrated satisfactory internal consistency at the scale level, parcels were constructed by grouping items into psychologically meaningful subdimensions consistent with the original factor structures. This approach preserves construct-level interpretation while ensuring statistical stability within the SEM framework. Importantly, parcels were not created arbitrarily. They reflect theoretically grounded psychological components underlying each construct (e.g., threat anticipation, attentional regulation, cognitive worry). Therefore, the measurement model captures process-level mechanisms rather than merely aggregating items for statistical convenience.

### 2.4 Procedure

Prior to data collection, ethical approval for the study was obtained from the Ethics Committee of Afyon Kocatepe University, Social and Human Sciences Scientific Research and Publication Ethics Board (Approval No: 2024/264). The study was conducted in accordance with the ethical principles outlined in the Declaration of Helsinki. The participants were informed that their participation was voluntary, that no personally identifiable information would be collected, and that all data would be used solely for scientific purposes in accordance with confidentiality principles. Written informed consent was obtained from all participants prior to participation.

Data were collected between March and April 2025 through an online survey administered via *Google Forms*. Participants were recruited using a convenience sampling method from high schools located in the city center of Afyon, Türkiye. Students were invited to participate by their teachers, who shared the survey link during school activities. In total, 1,100 students were reached, of whom 834 agreed to participate, yielding a response rate of 75.8%. Participation was entirely voluntary, and the students were informed that they could withdraw from the study at any time without providing a reason.

A non-probability convenience sampling strategy was employed due to institutional and administrative constraints inherent in school-based research settings. Access to participants required coordination with school administrations and teachers, limiting the feasibility of implementing probability-based sampling procedures. Although convenience sampling may restrict strict probabilistic representativeness, the relatively large and heterogeneous sample across multiple school types enhanced variability and supported the external validity of the findings.

### 2.5 Data analysis

Before the statistical analyses were conducted, the normality of the distributions for the main study variables was assessed. Skewness and kurtosis values were examined to evaluate univariate normality. According to Tabachnick and Fidell (2013), values between −1.5 and +1.5 are considered indicative of acceptable normality. The skewness and kurtosis values were found to be −0.153 and −0.040 for IU, 0.130 and 0.545 for mindfulness, and 0.032 and −0.370 for test anxiety, respectively. These results indicated that the variables were normally distributed.

In addition to univariate normality, multivariate normality was assessed using Mardia’s coefficient of multivariate kurtosis. The critical ratio (15.04) indicated deviation from strict multivariate normality. Given the large sample size (*N* = 834), maximum likelihood estimation was retained, as it is robust to moderate deviations from normality. To further evaluate model robustness, a Bollen–Stine bootstrap procedure with 2,000 resamples was conducted. Although the exact fit test was statistically significant (*p* < 0.001), approximate fit indices remained within recommended thresholds, supporting model stability.

Independent samples *t*-tests were conducted as descriptive analyses to examine potential gender-based differences in the study variables and to provide contextual information about their distribution across gender groups. These analyses were not incorporated into the structural equation model. Pearson correlation analysis was used to assess bivariate relationships among the variables.

The adequacy of the sample size for structural equation modeling was evaluated according to established SEM guidelines. With 834 participants and a parsimonious three-factor measurement model, the sample substantially exceeded recommended participant-to-parameter ratios. Additionally, the Critical N (CN) value exceeded the commonly recommended threshold of 200, indicating sufficient statistical power and stable parameter estimation. To test the primary hypothesis of the study—namely, the mediating role of mindfulness in the relationship between IU and test anxiety—a two-step structural equation modeling (SEM) procedure was conducted.

Indicator parceling was employed in the measurement model to improve indicator reliability, reduce random measurement error, and obtain more stable parameter estimates. Given that each scale demonstrated satisfactory internal consistency and theoretically coherent factor structures, items were combined into three balanced parcels per construct using the item-to-construct balance approach. Parceling enhances model parsimony while preserving construct-level interpretation (Little et al., 2002).

In the first step, the measurement model was evaluated to assess how well the observed variables represented their respective latent constructs. Model fit was evaluated via a comprehensive set of indices in line with recommended guidelines (Hu and Bentler, 1999; Kline, 2016). In addition to global fit indices, construct reliability and validity were assessed. Construct reliability and convergent validity were evaluated using composite reliability (CR) and average variance extracted (AVE). Discriminant validity was examined using the Fornell–Larcker criterion. Multicollinearity was assessed through examination of latent correlations and regression diagnostics, including variance inflation factor (VIF) and tolerance values.

Model fit was evaluated using multiple indices, including the root mean square error of approximation (RMSEA) with 90% confidence intervals and p-close statistic, the standardized root mean square residual (SRMR), and incremental fit indices such as the comparative fit index (CFI) and Tucker–Lewis index (TLI). Model stability was further examined using the expected cross-validation index (ECVI) and critical N (CN). Threshold values for acceptable and excellent fit followed established recommendations (e.g., CFI/TLI ≥ .95, RMSEA ≤ 0.06, SRMR ≤ 0.08). In the second step, the structural model was tested to examine the hypothesized relationships among the latent variables. The statistical analyses were conducted using SPSS version 27 and LISREL version 8.8.

## 3 Results

Independent samples *t*-tests were conducted to examine whether IU, mindfulness, and test anxiety differed by gender (Table 2). Female students scored significantly higher on intolerance of uncertainty than male students [*t*(832) = 4.29, *p* < 0.001, *d* = 0.33], indicating a small-to-moderate effect. Male students reported significantly higher levels of mindfulness than female students [*t*(832) = −3.12, *p* = 0.002, *d* = −0.24], reflecting a small effect. In contrast, male students demonstrated significantly higher test anxiety than female students [*t*(832) = −6.05, *p* < 0.001, *d* = −0.47], corresponding to a moderate effect size. These findings suggest that gender differences emerged particularly in uncertainty sensitivity and evaluative distress, although effect sizes remained within the small-to-moderate range.

**TABLE 2:** Independent samples *t*-test results for intolerance of uncertainty, mindfulness, and test anxiety by gender.

Variable	Groups	*n*	*M*	*SD*	*t*	*df*	*p*	Cohen’s *d*
Intolerance of uncertainty	Female	599	38.67	9.30	4.29	832	0.001***	0.33
Mindfulness	Male	235	35.54	9.97	−3.12	832	0.002**	−0.24
	Female	599	52.74	12.69				
Test anxiety	Male	235	55.85	13.59	−6.05	832	0.001***	−0.47
	Female	599	30.08	9.58				
	Male	235	34.54	9.58				

**p < 0.01;

***p < 0.001. All tests were two-tailed.

Pearson correlation analyses (Table 3) showed a coherent pattern of associations among the study variables. IU was negatively correlated with mindfulness (*r* range = −0.22 to −0.31, all *p* < 0.01), indicating that adolescents with lower tolerance for uncertainty tended to report diminished present-moment attentional awareness. IU was positively associated with test anxiety (*r* range = 0.28–0.42, all *p* < 0.01), suggesting that heightened sensitivity to ambiguity corresponds with greater evaluative distress. Mindfulness, in turn, was negatively correlated with test anxiety (*r* range = −0.36 to −0.49, all *p* < 0.01). These bivariate associations preliminarily supported a regulatory interpretation: adolescents who struggle with uncertainty appear more vulnerable to exam-related anxiety, whereas those with stronger attentional regulation capacities report lower distress.

**TABLE 3:** Pearson correlation coefficients for intolerance of uncertainty, mindfulness, and test anxiety.

Variable	INP1	INP2	INP3	MINP1	MINP2	MINP3	TAP1	TAP2	TAP3
INP1	1	1	1	1	1	1	1	1	1
INP2	0.624**								
INP3	0.565**	0.670**							
MINP1	−0.240**	−0.226**	−0.314**						
MINP2	−0.227**	−0.187**	−0.260**	0.675**					
MINP3	−0.249**	−0.230**	−0.291**	0.634**	0.667**				
TAP1	0.285**	0.286**	0.393**	−0.448**	−0.386**	−0.436**			
TAP2	0.300**	0.324**	0.402**	−0.404**	−0.363**	−0.442**	0.730**		
TAP3	0.324**	0.354**	0.424**	−0.445**	−0.388**	−0.488**	0.704**	0.737**	

**The correlation is significant at the *p* < 0.01 level (two-tailed). INP1– INP3 refer to the parcels of the intolerance of uncertainty variable; MINP1–MINP3 refer to the parcels of the mindfulness variable; and TAP1–TAP3 refer to the parcels of the test anxiety variable.

### 3.1 Measurement model

The measurement model was evaluated within the structural equation modeling framework to determine whether each latent construct was adequately represented by its parcels. All factor loadings were statistically significant and substantial. Intolerance of uncertainty (IU) was represented by three parcels (λ = 0.73–0.83), mindfulness by three parcels (λ = 0.80–0.82), and test anxiety by three parcels (λ = 0.84–0.86). These values indicated strong associations between the latent constructs and their observed indicators.

Beyond statistical adequacy, the parcels demonstrated psychological coherence consistent with the theoretical definitions of the constructs. The IU indicators (INP1–INP3) reflected threat anticipation and inhibitory responding under ambiguity, corresponding to heightened sensitivity to uncertain evaluative contexts. The mindfulness parcels (MINP1–MINP3) represented present-focused attentional stability and cognitive flexibility, capturing individuals’ tendency to maintain awareness of ongoing experience without excessive cognitive fusion. The test anxiety parcels (TAP1–TAP3) reflected cognitive worry, physiological arousal, and impaired concentration during evaluative situations, corresponding to the multidimensional experience of exam-related distress.

Squared standardized loadings (λ^2^) ranged from 0.53 to 0.69 for IU, 0.64 to 0.67 for mindfulness, and 0.71–0.74 for test anxiety, indicating that a substantial proportion of indicator variance was accounted for by their respective latent constructs. Composite reliability values were 0.83 for IU, 0.85 for mindfulness, and 0.89 for test anxiety, exceeding the recommended 0.70 criterion. Average variance extracted (AVE) values were 0.62, 0.66, and 0.72, respectively, surpassing the 0.50 threshold and supporting convergent validity. Discriminant validity was evaluated using the Fornell–Larcker criterion, and the square roots of AVE exceeded inter-construct correlations, indicating empirical distinctiveness among the latent constructs.

**FIGURE 1:**
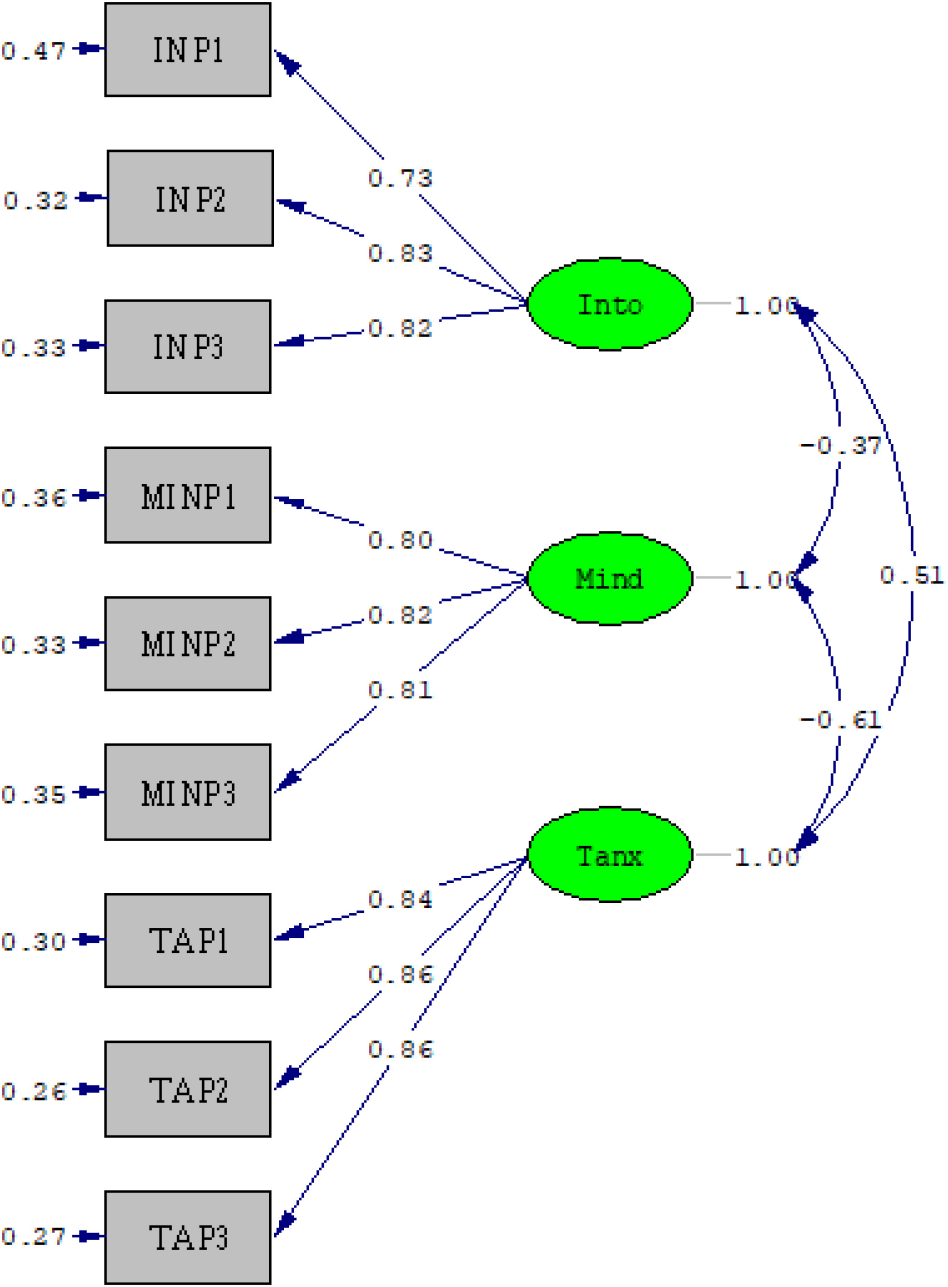
Measurement model representing the latent constructs of intolerance of uncertainty, mindfulness, and test anxiety. Into, intolerance of uncertainty; Mind, mindfulness; Tanx, test anxiety. The diagram was generated in LISREL 8.8 based on the confirmatory factor analysis results.

The overall measurement model demonstrated good fit to the data: χ^2^(24) = 74.44, *p* < 0.001; χ^2^/df = 3.10; RMSEA = 0.050 [90% CI (0.038, 0.063)]; SRMR = 0.031; CFI = .99; TLI = 0.99; NFI = 0.99. Although the chi-square statistic was significant, this is common among large samples. Collectively, these indices indicated an excellent correspondence between the hypothesized measurement structure and the observed data. As illustrated in Figure 1, each latent construct was strongly represented by its theoretically grounded parcels within the measurement framework.

### 3.2 Structural model and mediation analysis

**FIGURE 2:**
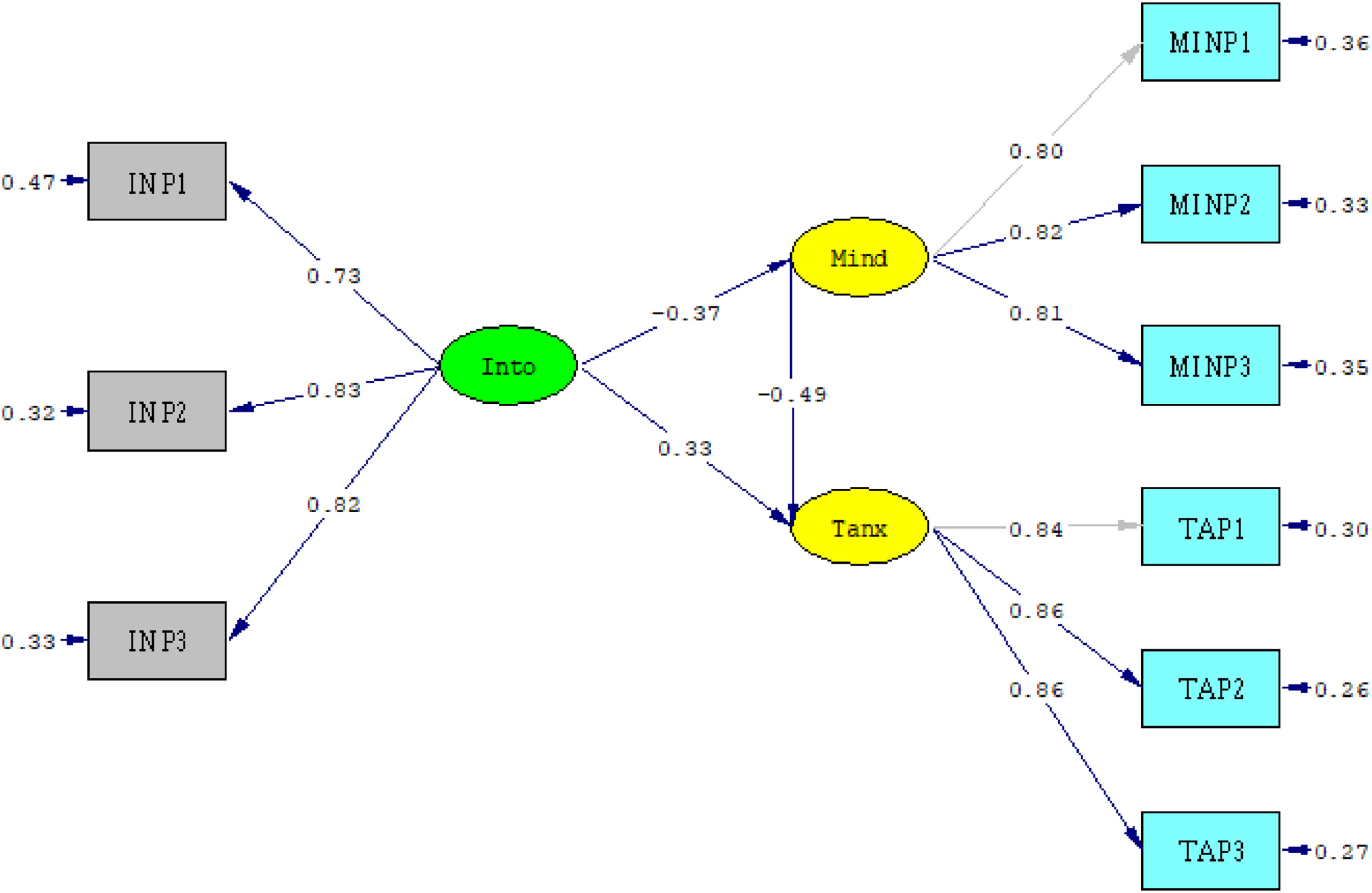
Structural model illustrating the relationships among intolerance of uncertainty, mindfulness, and test anxiety. Into, intolerance of uncertainty; Mind, mindfulness; Tanx, test anxiety. The diagram was generated in LISREL 8.8 based on the confirmatory factor analysis results.

Following validation of the measurement model, the structural model was tested to examine the mediating role of mindfulness in the association between IU and test anxiety (Figure 2).

The structural configuration reflects a partial mediation pattern in which IU is associated with both mindfulness and test anxiety, and mindfulness is also associated with test anxiety. IU was negatively and significantly associated with mindfulness (β = −0.37, SE = 0.041, *t* = −9.15, *p* < 0.001), accounting for 14% of its variance (*R*^2^ = 0.14). This result indicated that higher levels of intolerance of uncertainty were related to lower levels of present-focused attentional regulation.

Mindfulness was negatively and significantly associated with test anxiety (β = −0.49, SE = 0.039, *t* = −12.59, *p* < 0.001). Adolescents reporting higher levels of attentional stability and cognitive flexibility also tended to report lower levels of evaluative distress, including reduced cognitive worry and physiological activation. The model accounted for 47% of the variance in test anxiety (*R*^2^ = 0.47), indicating substantial explanatory capacity.

The direct path from IU to test anxiety remained positive and statistically significant (β = 0.33, SE = 0.042, *t* = 12.62, *p* < 0.001), supporting a partial mediation structure. Therefore, intolerance of uncertainty was associated with test anxiety both directly and indirectly through its association with mindfulness.

The indirect effect of IU on test anxiety through mindfulness was calculated as (−0.37 × −0.49) = 0.18. This positive indirect effect indicated that higher levels of IU were associated with higher levels of test anxiety via lower levels of mindfulness. In statistical terms, mindfulness serves as an intervening variable linking IU and test anxiety within the structural framework. The total effect of IU on test anxiety was 0.51 and statistically significant.

Taken together, the results support a partial mediation model in which intolerance of uncertainty is related to test anxiety both directly and indirectly through its association with attentional regulation capacities. The pattern of associations depicted in Figure 2 is consistent with a process-oriented interpretation of the relationships among dispositional uncertainty sensitivity, attentional regulation, and evaluative distress, while remaining within the limits of correlational structural modeling.

## 4 Discussion

The present study examined the mediating role of mindfulness in the relationship between intolerance of uncertainty (IU) and test anxiety within a high-stakes academic context. The study explored how individuals’ cognitive and emotional dispositions toward uncertainty are associated with test anxiety through attentional regulation and engagement with internal experiences. Previous research has demonstrated that mindfulness-based approaches are associated with reductions in affect intolerance and anxiety-related outcomes (Kraemer et al., 2020) and that mindfulness is negatively related to test anxiety (Yılmazer et al., 2024). However, limited research has integrated intolerance of uncertainty and mindfulness within a single structural mediation framework specifically targeting test anxiety in evaluative, high-stakes settings. By integrating these constructs into a single structural model, the present study addressed this empirical gap concerning how dispositional uncertainty sensitivity is associated with evaluative distress through attentional regulation processes. Rather than conceptualizing mindfulness as merely a correlate of anxiety—or solely as an intervention outcome or correlational predictor—the model positions mindfulness as a process-level regulatory pathway linking uncertainty sensitivity to exam-related distress, thereby advancing a process-oriented psychological framework for understanding test anxiety.

All four hypotheses were supported. IU was positively associated with test anxiety and negatively associated with mindfulness, whereas mindfulness was inversely associated with test anxiety. Importantly, mindfulness partially mediated the IU–test anxiety relationship. The persistence of a significant direct effect indicates that attentional regulation constitutes one pathway linking uncertainty sensitivity to evaluative distress, while additional cognitive mechanisms—such as catastrophic interpretations, negative performance expectations, or rigid threat appraisal—may also contribute to this association.

In addition to the structural relationships examined in the research model, the present study also showed several gender-based differences in the study variables. The findings indicated that female students reported significantly higher levels of intolerance of uncertainty, whereas male students demonstrated higher levels of mindfulness and test anxiety. However, the observed effect sizes ranged from small to moderate, suggesting that the influence of gender on the study variables was relatively limited.

The literature on intolerance of uncertainty indicates that this construct represents an important component of cognitive processes associated with anxiety and worry. It has been suggested that females may display greater cognitive sensitivity to uncertain situations in some samples, which has been associated with a higher tendency toward rumination and increased vulnerability to anxiety-related processes (Nolen-Hoeksema, 2012). Similarly, some studies assessing intolerance of uncertainty have reported that females tend to obtain higher IU scores than males (Buhr and Dugas, 2002). Research conducted with child and adolescent samples has also demonstrated that intolerance of uncertainty is closely associated with anxiety processes and that girls may report higher IU levels in specific analyses (Fialko et al., 2012). In this context, the finding that female students reported higher levels of intolerance of uncertainty in the present study appears to be consistent with some previous findings in the literature.

In contrast, studies in the mindfulness literature generally report that gender differences in dispositional mindfulness are limited or inconsistent (Baer et al., 2008; Brown and Ryan, 2003). Therefore, the higher mindfulness scores observed among male students in the present study may reflect a sample-specific variation. With regard to test anxiety, numerous studies have reported higher anxiety levels among female students (Putwain and Daly, 2014). However, the prevalence and severity of test anxiety may also be influenced by contextual factors such as academic performance pressure, the high-stakes nature of examinations, and characteristics of the educational system (von der Embse et al., 2018). Accordingly, the higher levels of test anxiety reported by male students in the present study may be related to sample-specific academic stress dynamics.

To further clarify how these structural relationships are reflected at the measurement level, the contributions of specific observed indicators were examined. Beyond latent-level associations, the strength of factor loadings suggested that particular psychological components were meaningfully embedded within the mediation pattern. Parcels reflecting discomfort with ambiguity and heightened threat anticipation were central within the IU construct, indicating that intolerance of uncertainty is closely tied to anticipatory cognitive activation under evaluative ambiguity. Mindfulness parcels representing attentional awareness and present-moment engagement were strongly associated with the mediating pathway, suggesting that regulatory attentional processes are closely linked to the IU–test anxiety association. Similarly, test anxiety parcels capturing cognitive worry and physiological arousal demonstrated robust representation, indicating that the mediation pattern corresponds not only to global distress but to specific experiential components of evaluative anxiety.

Beyond statistical associations, the findings gain greater clarity when interpreted in light of students’ lived psychological experiences during examinations. Adolescents high in IU may enter an examination setting with heightened vigilance toward uncertainty-related cues, such as ambiguous question wording or temporary retrieval difficulty. When encountering unfamiliar items, evaluative thoughts may escalate rapidly (e.g., *“If I cannot solve this, I will fail”*), accompanied by narrowing of attentional focus. This cognitive constriction may manifest as mental blankness, repetitive checking, slowed problem-solving, or avoidance of difficult questions. Simultaneously, physiological arousal—such as increased heart rate or muscle tension—may intensify threat-focused processing. As attentional resources shift toward monitoring uncertainty rather than engaging with task-relevant information, performance efficiency may decline. Within this context, the mediational pathway identified in the present model can be understood as reflecting observable moment-to-moment attentional disruptions during high-pressure evaluations.

These dynamics can be theoretically interpreted through the integration of Attentional Control Theory and Acceptance and Commitment Therapy. Attentional Control Theory posits that anxiety reduces the efficiency of goal-directed attentional systems by increasing stimulus-driven processing toward threat cues (Derakshan and Eysenck, 2009; Eysenck et al., 2007). High IU may heighten sensitivity to uncertainty signals during examinations, increasing attentional capture by perceived risk indicators such as time pressure or perceived knowledge gaps. The negative association between mindfulness and test anxiety suggests that present-focused attentional regulation may counterbalance this shift, allowing students to redirect cognitive resources toward task-relevant processing.

From an ACT perspective (Hayes et al., 1999), psychological distress is maintained not primarily by the presence of negative thoughts but by rigid, avoidant responses to them. Students high in IU may attempt to suppress or control uncertainty-related thoughts (*“I must not think about failing”*), thereby engaging in experiential avoidance. Such efforts may paradoxically intensify cognitive preoccupation and weaken attentional flexibility. Within this integrated framework, mindfulness represents a regulatory capacity that enables individuals to experience uncertainty-related cognitions without escalating evaluative reactivity. By combining attentional control mechanisms with experiential flexibility processes, the present findings support a theoretically integrated model of test anxiety in academic contexts.

The theoretical contribution of the study lies in extending both frameworks into a high-stakes academic performance domain. While Attentional Control Theory has predominantly been examined in laboratory-based anxiety paradigms and ACT in clinical populations, the present results suggest that their combined explanatory value may illuminate regulatory processes operating in everyday educational settings. Rather than conceptualizing test anxiety solely as a reaction to external evaluative pressure, the findings support a process-oriented interpretation in which evaluative distress is associated with the interaction between dispositional uncertainty sensitivity and reduced attentional–experiential regulation capacities.

## 5 Limitations

Several limitations should be considered when interpreting these findings. First, the cross-sectional design limits conclusions regarding temporal ordering among variables. The mediation pattern represents covariance-based structural relationships rather than confirmed causal sequencing. Longitudinal or cross-lagged designs are needed to examine temporal precedence and potential reciprocal influences among IU, mindfulness, and test anxiety.

Second, all constructs were assessed via self-report measures, raising the possibility of shared method variance and response bias. Future research incorporating behavioral attentional tasks or physiological indicators (e.g., heart rate variability during examination simulations) would strengthen multimethod validity.

Third, although parceling enhanced parsimony and structural stability, replication at the item level would provide a more granular examination of indicator-specific dynamics. Additionally, alternative mediating or moderating variables—such as cognitive distortions, academic self-efficacy, or safety-seeking behaviors—were not examined and may further clarify the structural pathways.

Finally, the Turkish educational system’s emphasis on centralized high-stakes examinations may heighten uncertainty salience. Replication across diverse cultural and educational contexts is necessary to determine the generalizability of the integrated attentional–experiential framework.

## 6 Recommendations

The findings suggest several implications for educational practice and future intervention research. Screening for elevated intolerance of uncertainty may help school counselors identify students at greater risk for evaluative anxiety escalation. Brief attentional reset exercises—such as structured 2–5-min breath-focused grounding practices before mock examinations—may help students rehearse shifting attention away from intrusive uncertainty-related cognitions.

Psychoeducational modules that normalize uncertainty as an inherent feature of evaluative environments may reduce catastrophic interpretations. ACT-informed small-group interventions focusing on cognitive defusion and values-based engagement may strengthen psychological flexibility. Rather than attempting to eliminate anxiety, such programs may aim to enhance students’ capacity to sustain task-focused attention despite uncertainty-related thoughts. Future research should employ longitudinal and experimental designs to examine whether attentional control training or ACT-based interventions produce measurable reductions in test anxiety, particularly among students with elevated IU.

## 7 Conclusion

The present study demonstrated that mindfulness partially mediates the relationship between intolerance of uncertainty and test anxiety within a high-stakes academic context, while IU also maintains a significant direct association with evaluative distress. These findings suggest that test anxiety is associated not only with external performance demands but also with internal regulatory processes involving attentional control and experiential flexibility. By integrating perspectives from Attentional Control Theory and Acceptance and Commitment Therapy, the study provides a theoretically grounded, process-oriented framework for understanding how uncertainty-related cognitive and affective tendencies are linked to evaluative anxiety in academic settings. Strengthening attentional regulation and psychological flexibility may therefore represent promising targets for preventive efforts in educational contexts.

## Data Availability

The raw data supporting the conclusions of this article will be made available by the authors, without undue reservation.
